# Whole Genome Sequencing identifies reciprocal translocation *hT2(I;III)* breakpoints.

**DOI:** 10.17912/micropub.biology.000505

**Published:** 2021-12-09

**Authors:** Stephane Flibotte, Mark Edgley, Vinci Au, Donald G Moerman

**Affiliations:** 1 UBC/LSI Bioinformatics Facility, University of British Columbia, Vancouver, British Columbia, Canada; 2 Department of Zoology, University of British Columbia, Vancouver, British Columbia, Canada

## Abstract

We used whole-genome sequencing (WGS) data from a *Caenorhabditis elegans* strain homozygous for the reciprocal translocation *hT2(I;III) *to identify its breakpoints molecularly. The translocation structure is fairly straightforward, with only minor secondary rearrangement in addition to the primary breakpoints. The graphical representation below depicts the two *hT2* half-translocations for ease of conceptualization.

**Figure 1. Chromosomes I and III in the hT2 balancer:**
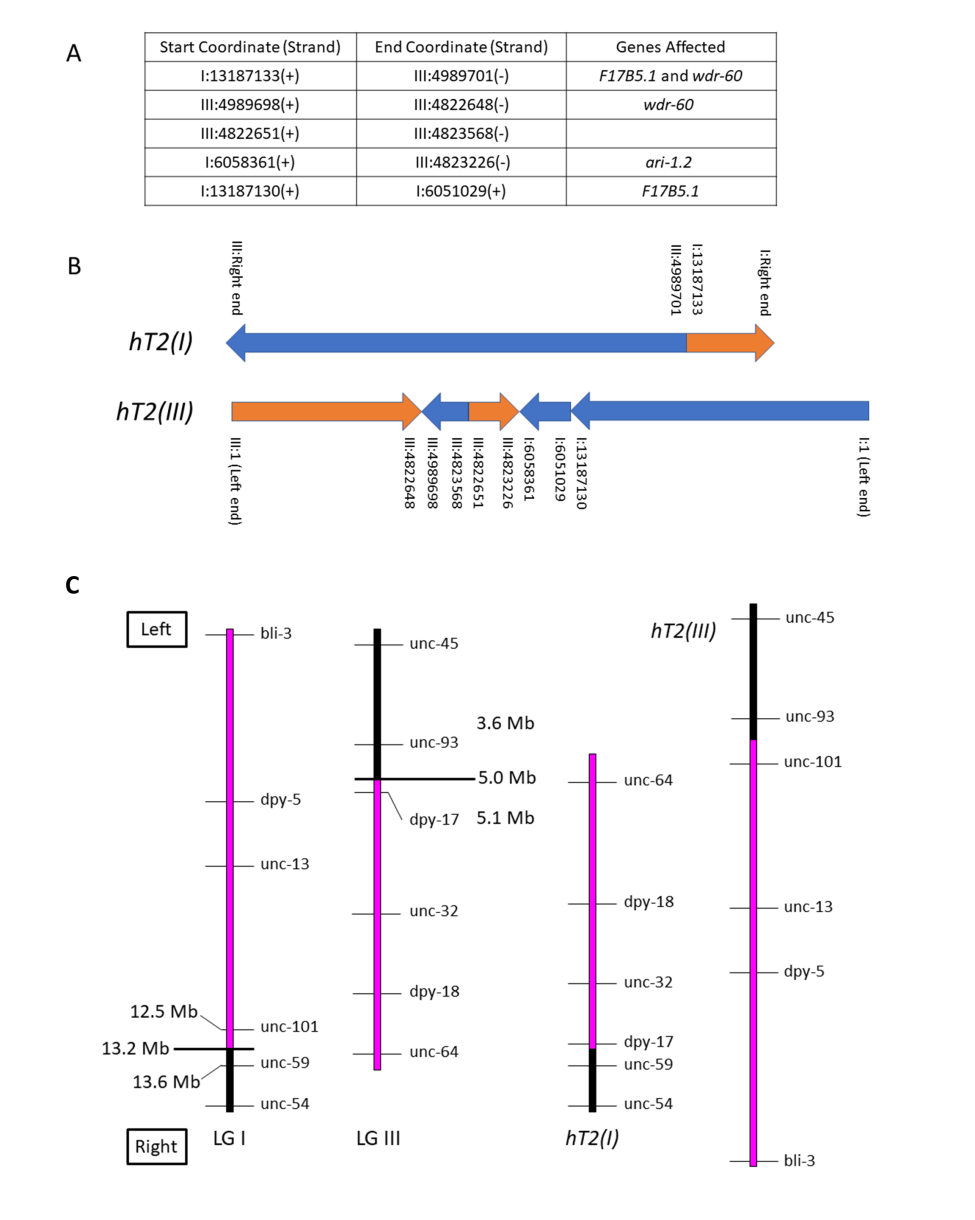
(**A**) Breakpoint connections deduced by aligning split reads at the breakpoints. Identical strands indicates that the sequence on the left is connected directly to the sequence on the right, while different strands denotes a change of strand. Genes affected by the breakpoints are listed in the third column. (**B**) A schematic representation of chromosomes I and III deduced for *hT2* is shown. Each arrow represents the chromosomal segment of the reference sequence with the corresponding coordinates listed. The direction and color of each arrow represent the strand/direction of the sequence within the original reference chromosome. (**C**) Simplified representation (not at perfect scale) of wild-type (left two chromosomes) and the *hT2* translocation (right two chromosomes) with standard marker genes indicated. Chromosome regions in magenta represent segments that are translocated in *hT2*, while those in black represent untranslocated segments responsible for correct meiotic pairing. For the wild-type chromosomes, the lines at 13.2 Mb (LG I) and 5.0 Mb (LG III) indicate approximate positions of translocation breakpoints from WGS data.

## Description

The reciprocal translocation *hT2* was isolated after gamma irradiation of *bli-4(e937)I* males followed by crossing to *unc-13(e450) I; dpy-18(e364) III* hermaphrodites and screening F2 broods for evidence of pseudolinkage (McKim *et al.*, 1993). Subsequent genetic experiments, including variants of *hT2* with new morphological mutations induced by mutagenesis, elucidated its genetic structure as a reciprocal translocation and its meiotic pairing and segregation behavior. It is comprised of two half-translocations: *hT2(I),* which pairs with and disjoins from the normal LG I in *hT2* heterozygotes; and *hT2(III)*, which pairs with and disjoins from the normal LG III in heterozygotes. In simple terms, the mutagenesis event that produced *hT2* cleaved LG I between *unc-101* and *unc-59*, and LG III between *dpy-17* and *unc-93*; in *hT2*, the resulting rightmost portion of LG III is translocated to the rightmost portion of LG I to form *hT2(I),* and the leftmost portion of LG I is translocated to the leftmost portion of LG III to form *hT2(III)* (see Figure 1).

The half-translocation *hT2(I)* runs from the right end of LG I through coordinate 13187133, where it joins to the right portion of LG III from coordinate 4989701 through the right end. The half-translocation *hT2(III)* runs from LG III coordinate 1 through coordinate 4822648, where it joins to a short complex rearrangement containing pieces of both LG I and LG III, and then to a portion of LG I from coordinate 1 through 13187130. These physical breakpoints correspond to the genetic limits of balancing behavior observed in recombination analysis of marker/*hT2* *trans* heterozygotes. Such experiments showed that *unc-101* (I: 12508299 – 12513420) is balanced, while *unc-59* (I: 13629227 – 13633492) is not; and *dpy-17* (III: 5107330 – 5108492) is balanced, while *unc-93* (III: 3644375 – 3648822) is not.

*hT2* has been used extensively as an effective balancer for the left portion of LG I from the left end through *unc-101*, and right portion of LG III from the right end through *dpy-17* (see Figure 1). The original *hT2* is homozygous viable and is marked with *bli-4(e937)I*, and several morphological variants exist (summarized in Edgley *et al.*, 1995). The CGC maintains over 450 strains that utilize variations of this balancer for maintaining mutant alleles of essential genes.

## Methods

**Sequence Generation**

A large asynchronous population of *hT2[bli-4(e937)](I;III)*, established from a single Bli-4 segregant from VC386 (*nmy-2(ok499)/hT2 I; +/hT2 [bli-4(e937)] III)* was harvested by washing freshly-starved 100 mm standard agar/OP50 culture plates with M9 buffer. Worms were pelleted in 15 ml centrifuge tubes, the supernatant was removed by aspiration, and the packed worms were used to make purified DNA using standard extraction protocols. The sequencing library was made using Illumina’s Nextera XT library prep kit and run on an Agilent Bioanalyzer to check average fragment size. The sequencing was performed with Illumina MiSeq using 2 x 300 bp reads and an average coverage of 24X was acquired.

**Sequence Analysis**

The raw sequence data from this study have been deposited in the NCBI Sequence Read Archive (SRA; ncbi.nlm.nih.gov/sra) under accession number PRJNA780557. The sequencing reads were first aligned to the reference *C. elegans* genome version WS230 using the BWA aligner (Li and Durbin 2009). Read alignments were visually inspected in the regions of chromosomes I and III expected to contain the breakpoints associated with hT2 translocation using the IGV genome viewer (Thorvaldsdóttir *et al*., 2013). LUMPY (Layer *et al.*, 2014) was also used to search for potentially missed breakpoints from the visual inspection. Each breakpoint was then further analyzed by aligning split reads at the breakpoint using the blast tool available on the WormBase website (version WS282, https://wormbase.org/tools/blast_blat), which allowed connecting two breakpoints to one another as reported in **Fig. 1A**. Mutated chromosomes I and III for hT2 were then deduced using those breakpoint connections and a parsimonious approach while taking into account the copy-number evidence provided by the read coverage in the regions of interest.

## Reagents

DNA sequence analysis was based on the wild type N2 Bristol derived strain PD1074 and a Bli *hT2* homozygous derivative of strain VC386 *nmy-2(ok499)/hT2 I; +/hT2 [bli-4(e937)].*
